# In Silico Exploration of Metabolically Active Peptides as Potential Therapeutic Agents against Amyotrophic Lateral Sclerosis

**DOI:** 10.3390/ijms24065828

**Published:** 2023-03-18

**Authors:** Toluwase Hezekiah Fatoki, Stanley Chukwuejim, Chibuike C. Udenigwe, Rotimi E. Aluko

**Affiliations:** 1 Department of Biochemistry, Federal University Oye-Ekiti, PMB 373, Oye 371104, Nigeria; toluwase.fatoki@fuoye.edu.ng (T.H.F.); stanley.chukwuejim@fuoye.edu.ng (S.C.); 2 Department of Food and Human Nutritional Sciences, University of Manitoba, Winnipeg, MB R3T 2N2, Canada; 3 Faculty of Health Sciences, School of Nutrition Sciences, University of Ottawa, Ottawa, ON K1H 8M5, Canada; cudenigw@uottawa.ca; 4 Department of Chemistry and Biomolecular Sciences, Faculty of Science, University of Ottawa, Ottawa, ON K1N 6N5, Canada; 5 Richardson Centre for Food Technology and Research, University of Manitoba, Winnipeg, MB R3T 2N2, Canada

**Keywords:** amyotrophic lateral sclerosis, ALS, associated genes, hydrolysate peptides, pharmacokinetics, target prediction, metabolic, supplements, molecular docking

## Abstract

Amyotrophic lateral sclerosis (ALS) is regarded as a fatal neurodegenerative disease that is featured by progressive damage of the upper and lower motor neurons. To date, over 45 genes have been found to be connected with ALS pathology. The aim of this work was to computationally identify unique sets of protein hydrolysate peptides that could serve as therapeutic agents against ALS. Computational methods which include target prediction, protein-protein interaction, and peptide-protein molecular docking were used. The results showed that the network of critical ALS-associated genes consists of ATG16L2, SCFD1, VAC15, VEGFA, KEAP1, KIF5A, FIG4, TUBA4A, SIGMAR1, SETX, ANXA11, HNRNPL, NEK1, C9orf72, VCP, RPSA, ATP5B, and SOD1 together with predicted kinases such as AKT1, CDK4, DNAPK, MAPK14, and ERK2 in addition to transcription factors such as MYC, RELA, ZMIZ1, EGR1, TRIM28, and FOXA2. The identified molecular targets of the peptides that support multi-metabolic components in ALS pathogenesis include cyclooxygenase-2, angiotensin I-converting enzyme, dipeptidyl peptidase IV, X-linked inhibitor of apoptosis protein 3, and endothelin receptor ET-A. Overall, the results showed that AGL, APL, AVK, IIW, PVI, and VAY peptides are promising candidates for further study. Future work would be needed to validate the therapeutic properties of these hydrolysate peptides by in vitro and in vivo approaches.

## 1. Introduction

Amyotrophic lateral sclerosis (ALS) is one of the motor neuron diseases (MNDs) and is a fatal progressive neurodegenerative disease that affects both the lower and upper motor neurons, causing paralysis and ultimately leading to demise due to respiratory failure, usually between 3 to 5 years after the first symptoms [1]. ALS can occur sporadically (sALS) in people without any seeming family history or be genetically inherited (fALS) [2]. The general incidence of ALS in North America and Europe is projected to be 3–5 per 100,000 human population and increases within the ageing population, which is accompanied by the death of about 11,000 Europeans and 6000 Americans annually [3]. However, there is a scarcity of data that currently focuses on the African population, though existing reports suggest that the majority of African patients with ALS are male with a median age at onset of 53.0 years (interquartile range, IQR: 44.5–64.0 years) and that they exhibit a lesser proportion of bulbar onset compared with patients from western countries [4,5,6].

ALS is a complex disease because it has been linked to multiple aggregating proteins which include chromosome 9 open reading frame 72 (C9ORF72), superoxide dismutase 1 (SOD1), tar-DNA binding protein 43 (TDP-43), rho guanine nucleotide exchange factor (RGNEF), phosphorylated high molecular weight neurofilament protein (pNFH), fused in sarcoma (FUS), and others [7]. To date, over 45 genes have been found linked to ALS pathology [8], but there is still an inadequate understanding of the disease mechanisms. Efforts involving the discovery and validation of genes causal to ALS pathology have yielded critical insights into relevant cellular mechanisms that lead to neurodegeneration such as autophagy defects, cytoskeleton alterations, endoplasmic reticulum stress, nucleocytoplasmic transport, protein homeostasis, and RNA metabolism [1].

In the United States of America (USA), four drugs (Riluzole, Tiglutik, Edaravone, and Nuedexta) for the treatment of ALS have been approved by the Food and Drug Administration (FDA) [9]. Edaravone (a free radical scavenger) and riluzole (a glutamate release inhibitor) are used for the treatment of ALS through mechanisms that only delay disease progression and prolong survival for about 2–3 months [2,10]. In ALS patients, there is a tendency of difficulties in swallowing, an oral suspension of riluzole, branded as Tiglutik, has been recommended, while Nuedexta, an oral medication containing dextromethorphan and quinidine, could be used for lowering the severity and rate of the pseudobulbar effect [9,11]. Presently, there is no effective therapy for curing ALS. Moreover, it is evident from research that there exist links between metabolic component and ALS pathogenesis, thus providing the possibility of more molecular targets for therapeutic purposes [8,12]. However, the lack of effective treatment options leads many ALS patients to seek alternative therapies such as dietary supplements. In September 2022, the FDA approved a dietary supplement called Relyvrio (sodium phenylbutyrate-taurursodiol) to treat patients with ALS. The use of this supplement caused a greater delay in the functional decline than a placebo, as measured by the ALS Functional Rating Scale Revised (ALSFRS-R) score for a period of 24 weeks [13].

In ALS patients, there are signs of malnutrition and weight loss which are often due to progressive muscle atrophy, insufficient oral absorption of nutrients, and the potential hypermetabolic state that have been associated with quick disease progression [14]; thus, adequate calorie and protein intake is necessary. Considering the abnormalities in the complex metabolic processes implicated in ALS, normal and fiber-enriched dietary formulas would be inadequate to achieve nutritional support and optimal metabolic status [15]. To overcome common barriers that limit the consumption of adequate nutrition for ALS patients, the features of an effective therapeutic and dietary supplement include high calorie-based, liquid, less or zero fiber, probiotic and ozonized, appetite booster, and anti-depressant [12,16,17,18].

Dietary proteins contain essential amino acids that are necessary for the proper maintenance of the human body. Protein quality is the property of a protein to trigger specific metabolic outcomes [19], which vary based on the health status including disease state, medication use, and physical activity. Bioactive peptides (BAPs) are made up of short sequences of 2–10 amino acid residues produced by the proteolytic cleavage of proteins from biologically safe sources; BAPs often exhibit similar or unrelated biological properties when compared to the sample protein [20]. The biofunctional peptides derived from plants, animal, microorganisms, or through recombinant technology have been reported useful in treating or preventing specific diseases, disorders, and conditions linked to muscle wasting, as well as in improving and maintaining muscle health [21]. Loss of muscle mass primarily occurs in the skeletal muscle tissues when the rate of proteolysis exceeds that of protein synthesis [22]. Leucine has been linked to protein synthesis in the muscle, and protein sources such as whey provide adequate free amino acids for maintaining protein synthesis in the muscle [23,24].

Moreover, a study has found that supplementation of L-serine in cyanotoxin β-N-methylamino-L-alanine (BMAA)-induced ALS/MND-type pathological variations in the vervet model reduced the number of protein inclusions and the amount of reactive gliosis in motor neurons [25]. Additionally, a novel antioxidant cell-permeable peptide (CPP) SS-31 (D-Arg-Dmt-Lys-Phe-NH_2_, where Dmt = 2′,6′-dimethyltyrosine), which consists of alternating basic and aromatic amino acid residues with dimethyltyrosine, scavenged reactive oxygen species (ROS) in an ALS mouse model [26]. Meanwhile, a novel D-peptide RD2RD2 (PTLHTHNRRRRRPTLHTHNRRRRR, with an amidated C-terminus), classified as cationic arginine-rich CPP, was reported to reduce activated glia cell levels in the lumbar spinal cord and brain stem in SOD1^G93A^ mouse model of ALS [27]. Therefore, selective uptake of peptides by specific tissues and cells of interest could trigger the desired therapeutic outcome when dietary requirements for proteins are well defined to optimize the metabolic roles of amino acids and peptides [28]. Therefore, the aim of this work was to computationally identify the molecular targets of unique sets of protein hydrolysate peptides that could be used to formulate therapeutic supplements for ALS patients.

## 2. Results

**Figure 1:**
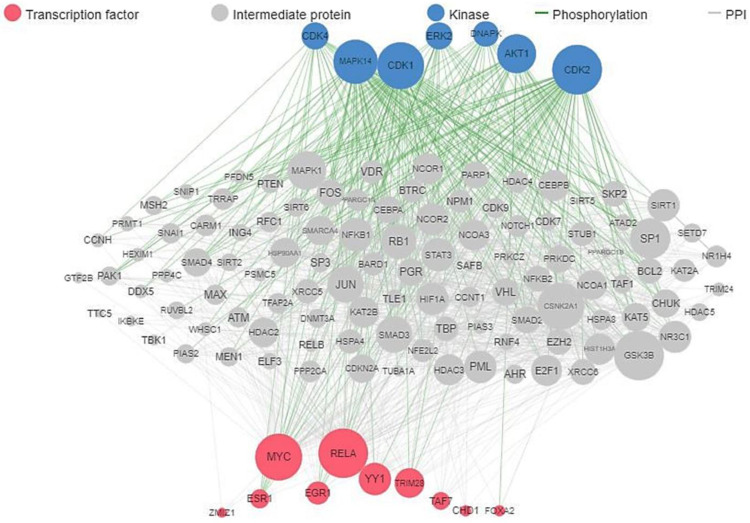
ALS published target gene network of kinases, protein-protein interactions, and transcription factors.

**Figure 2:**
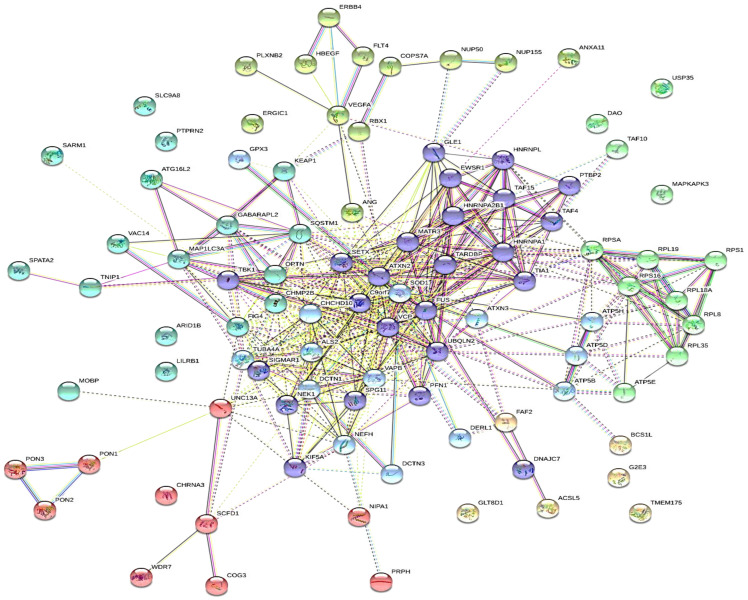
Protein-protein interactions of all the ALS-associated genes, visualized with k-mean clustering into seven clusters. Legend: Cluster 1: red (CHRNA3, COG3, NIPA1, PON1, PON2, PON3, PRPH, SCFD1, UNC13A, and WDR7). Cluster 2: brown (ACSL5, BCS1L, FAF2, G2E3, GLT8D1, and TMEM175). Cluster 3: olive (ANG, ANXA11, COPS7A, ERBB4, ERGIC1, FLT4, HBEGF, NUP155, NUP50, PLXNB2, RBX1, and VEGFA). Cluster 4: green (ATP5E, DAO, MAPKAPK3, RPL18A, RPL19, RPL35, RPL8, RPS12, RPS16, RPSA, TAF10, and USP35). Cluster 5: blue (ARID1B, ATG16L2, CHMP2B, FIG4, GABARAPL2, KEAP1, LILRB1, MAP1LC3A, MOBP, OPTN, PTPRN2, SARM1, SLC9A8, SPATA2, SQSTM1, TNIP1, and VAC14). Cluster 6: light sky blue (ALS2, ATP5B, ATP5D, ATP5H, ATXN3, CHCHD10, DCTN1, DCTN3, DERL1, GPX3, NEFH, SOD1, TUBA4A, and VAPB). Cluster 7: medium blue (ATXN2, C9orf72, DNAJC7, EWSR1, FUS, GLE1, HNRNPA1, HNRNPA2B1, HNRNPL, KIF5A, MATR3, NEK1, PFN1, PTBP2, SETX, SIGMAR1, SPG11, TAF15, TAF4, TARDBP, TBK1, TIA1, UBQLN2, and VCP). Dotted lines show inter-cluster interactions.

**Figure 3:**
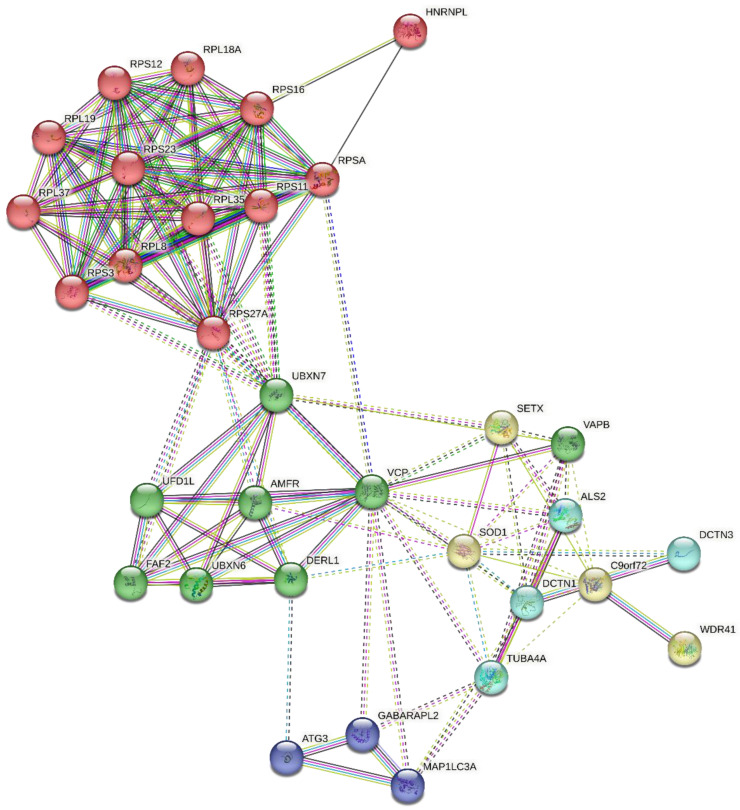
Threefold expanded protein-protein interactions of 12 selected ALS-associated genes (RPSA, MAP1LC3A, GABARAPL2, SOD1, ALS2, VAPB, TUBA4A, DCTN1, SETX, C9orf72, VCP, and HNRNPL), visualized with k-mean clustering into five clusters. Legend: Cluster 1: red (HNRNPL, RPL18A, RPL19, RPL35, RPL37, RPL8, RPS11, RPS12, RPS16, RPS23, RPS27A, RPS3, and RPSA). Cluster 2: yellow (C9orf72, SETX, SOD1, and WDR41). Cluster 3: green (AMFR, DERL1, FAF2, UBXN6, UBXN7, UFD1L, VAPB, and VCP). Cluster 4: cyan (ALS2, DCTN1, DCTN3, and TUBA4A). Cluster 5: blue (ATG3, GABARAPL2, and MAP1LC3A). Dotted lines show inter-cluster interactions.

The analysis of the existing network of 76 ALS-associated genes investigated in this work showed the involvement of MYC, RELA, YY1, TRIM28, EGR1, ESR1, ZMZ1, TAFT, CHD1, and FOXA2 transcription factors, and the kinases MAPK14, CDK1, CDK2, CDK4, DNAPK, and AKT1, as shown in Figure 1. Protein-protein interactions of all the ALS-associated genes, visualized with k-mean clustering into seven groups as shown in Figure 2 revealed the network of core genes that are closely associated with ALS disease. The DCTN1-KIF5A gene fusion in humans was predicted from the STRING protein-protein interactions. Figure 3 shows the threefold expanded protein-protein interactions of 12 selected ALS-associated genes (RPSA, MAP1LC3A, GABARAPL2, SOD1, ALS2, VAPB, TUBA4A, DCTN1, SETX, C9orf72, VCP, and HNRNPL) visualized with k-mean clustering into five clusters. The results excavated more sets of genes, which include WDR41, AMFR, DERL1, FAF2, and UFD1L, within cluster 3 where VAPB and VCP are present.

**Figure 4:**
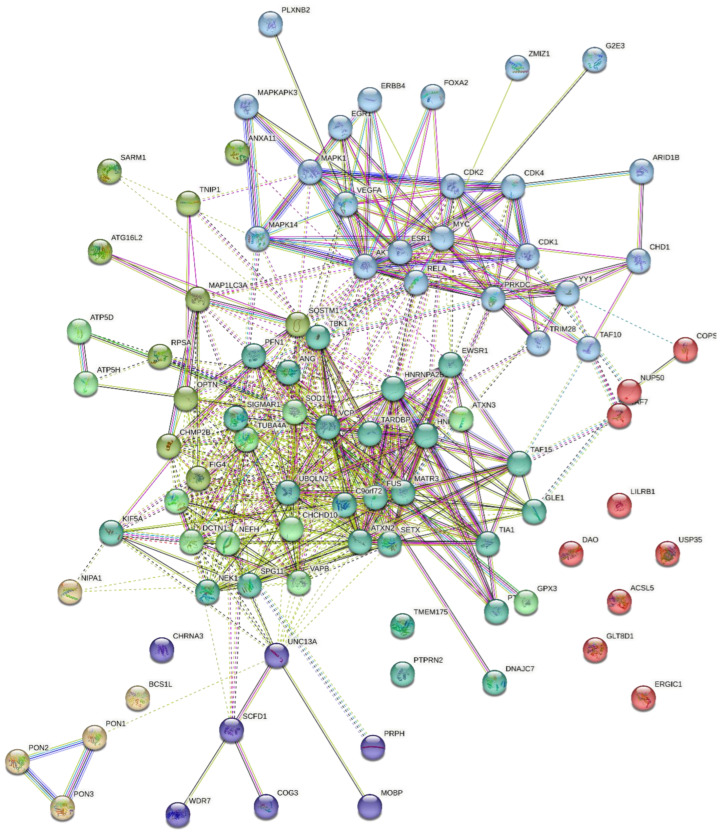
Protein-protein interaction network of all ALS-associated genes together with predicted kinases and transcription factors, visualized with k-mean clustering into seven clusters. Legend: Cluster 1: red (ACSL5, COPS7A, DAO, ERGIC1, GLT8D1, LILRB1, NUP50, TAF7, and USP35). Cluster 2: brown (BCS1L, NIPA1, PON1, PON2, and PON3). Cluster 3: olive (ANXA11, ATG16L2, CHMP2B, FIG4, MAP1LC3A, OPTN, RPSA, SARM1, SQSTM1, and TNIP1). Cluster 4: green (ALS2, ATP5D, ATP5H, ATXN3, CHCHD10, DCTN1, GPX3, NEFH, SOD1, TUBA4A, and VAPB). Cluster 5: blue (ANG, ATXN2, C9orf72, DNAJC7, EWSR1, FUS, GLE1, HNRNPA1, HNRNPA2B1, KIF5A, MATR3, NEK1, PFN1, PTBP2, PTPRN2, SETX, SIGMAR1, SPG11, TAF15, TARDBP, TBK1, TIA1, TMEM175, UBQLN2, and VCP). Cluster 6: light sky blue (AKT1, ARID1B, CDK1, CDK2, CDK4, CHD1, EGR1, ERBB4, ESR1, FOXA2, G2E3, MAPK1, MAPK14, MAPKAPK3, MYC, PLXNB2, PRKDC, RELA, TAF10, TRIM28, VEGFA, YY1, and ZMIZ1). Cluster 7: medium blue (CHRNA3, COG3, MOBP, PRPH, SCFD1, UNC13A, and WDR7). Dotted lines show inter-cluster interactions.

Figure 4 presents the protein-protein interaction network of all ALS-associated genes (ATG16L2, SCFD1, VAC15, VEGFA, KEAP1, KIF5A, FIG4, TUBA4A, SIGMAR1, SETX, ANXA11, HNRNPL, NEK1, C9orf72, VCP, RPSA, ATP5B, and SOD1) together with the predicted kinases (AKT1, CDK1, CDK2, CDK4, DNAPK, MAPK14, and ERK2) and transcription factors (MYC, RELA, ZMIZ1, ESR1 EGR1, YY1, TRIM28, TAF7, CHD1, and FOXA2) visualized with k-mean clustering into seven clusters. This result indicates clusters four and five which consist of core ALS genes are highly associated with cluster six that consists of transcription factors and kinases.

The in silico hydrolysis of the investigated peptides and proteins by simultaneous cleavage with chymotrypsin, trypsin, and pepsin yielded several peptides (Supplementary Table S1). Target prediction was then used to select the following 13 peptides with active molecular targets of at least 20% probability (Table 1): AGL and SGGVVK (from calcitonin gene-related peptide 1, seq. 83–119), APL (from Spadin), AVK (from vasoactive intestinal peptide, seq. 125–152), AVY (from LAVYPWT), IGF and IIW (from Peptide H3), PVI (from potato patatin-derived peptide), SDPF (from Phoenixin PNX-14, seq. 51–64), AIF and VAY (from GABARAPL2 peptide), TEL (from Substance P, seq.1–11), and APVSIPQ (from NAP peptide). The 24 molecular targets obtained which are relevant to ALS disease progression include: cyclooxygenase-2 (UniProt ID: P35354), angiotensin I-converting enzyme (UniProt ID: P12821), HLA class I histocompatibility antigen A-3 (UniProt ID: P04439), Dipeptidyl peptidase IV (UniProt ID: P27487), inhibitor of apoptosis protein 3 (UniProt ID: P98170), Mu opioid receptor (UniProt ID: P35372), delta opioid receptor (UniProt ID: P41143), disks large homolog 4 (UniProt ID:P78352), endothelin receptor ET-A (UniProt ID: P25101), and HMG-CoA reductase (UniProt ID: P04035).

**Figure 5:**
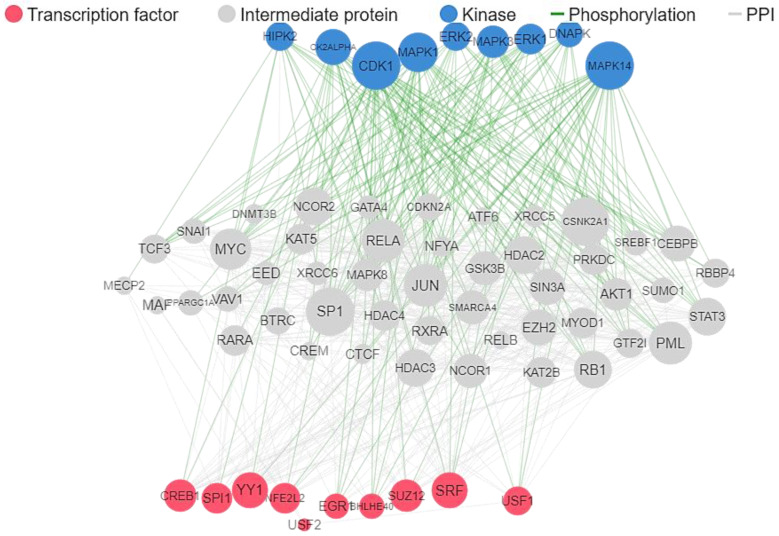
ALS predicted target gene network of kinases, protein-protein interactions, and transcription factors.

Overall, as shown in Table 1, AGL had the highest target probability for cyclooxygenase-2 (PTGS2), AIF for angiotensin converting enzyme (ACE), TEL and VAY for HLA class I histocompatibility antigen A-3 (HLA-A), PVI for dipeptidyl peptidase IV (DPP4) and inhibitor of apoptosis protein 3 (XIAP), AVY for Mu opioid receptor (OPRM1), IIW and AVY for endothelin receptor ET-A/ET-B (EDNRA/EDNRB), and SDPF for HMG-CoA reductase (HMGCR). The reconstructed protein-protein interaction network based on the active molecular targets with a least 20% probability (Figure 5) implicates CDK1, ERK1 (MAPK3), ERK2 (MAPK1), DNAPK, MAPK14, HIPK2, and CK2AALPHA kinases as well as SRF, EGR1, USF1, USF2, SUZ12, BHLHE40, NFE2L2, YY1, SPI1, and CREB1 transcriptional factors.

The results of predicted pharmacokinetics properties in Table 2 show that all the 13 peptides investigated in this work are very soluble, and not permeants of the blood-brain barrier (BBB). Furthermore, only AGL, AIF, APL, IGF, IIW, and PVI peptides have high gastrointestinal absorption (GIA) while IIW peptide could inhibit cytochrome P450 3A4 (CYP3A4), and APL, IIW, PVI, and SGGVVK peptides are -glycoprotein (P-gp) substrates. The bioavailability score of APVSIPQ, SDPF, and TEL peptides are very low (0.11–0.17) when compared to the others (0.55).

**Figure 6:**
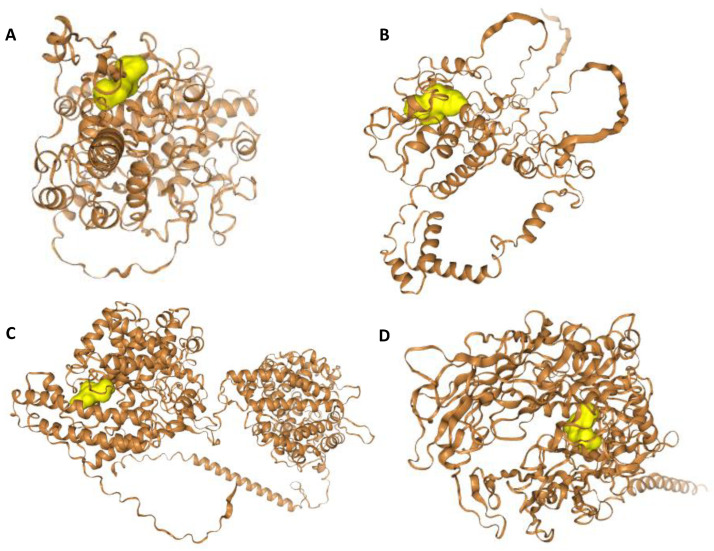
HPEPDOCK docking interaction of (**A**) peptide AGL with cyclooxygenase-2 (PTGS2, P35354), (**B**) peptide AIF with inhibitor of apoptosis protein 3 (XIAP, P98170), (**C**) peptide IIW with angiotensin I-converting enzyme (ACE, P12821), and (**D**) peptide PVI with dipeptidyl peptidase IV (DPP4, P27487).

**Figure 7:**
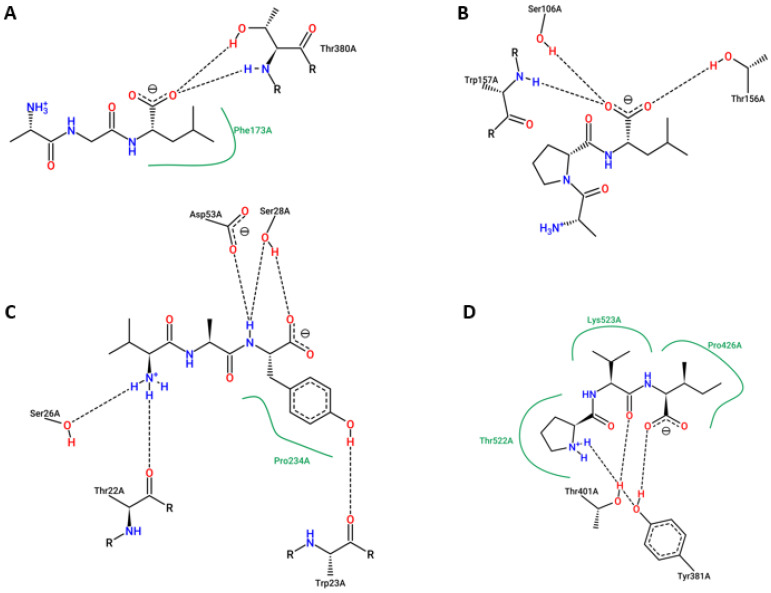
PoseView of AutoDock vina docking interaction of (**A**) AGL and cyclooxygenase-2 (PTGS2, P35354), (**B**) APL and dipeptidyl peptidase IV (DPP4, P27487), (**C**) VAY and HLA class I histocompatibility antigen A-3 (HLA-A, P04439), and (**D**) PVI and dipeptidyl peptidase IV (DPP4, P27487).

The results of HPEPDOCK molecular docking analyses showed that these peptides have good binding affinity to the protein targets (Table 3). The peptide IIW had an overall docking score of −188.427 against Mu opioid receptor (OPRM1, P35372), followed by −172.819 against ACE (P12821). Peptides AVY, PVI, and AGL have docking scores of −121.698 against inhibitor of apoptosis protein 3 (XIAP, P98170), −118.817 against DPP4 (P27487), and −91.468 against cyclooxygenase-2 (PTGS2, P35354), respectively. AutoDock Vina docking binding affinity score of the interactions of ligands (peptides) and selected predicted targets with at least 40% probability (Table 3) shows the highest value of −7.912 kcal.mol^−1^ for interaction of IIW peptide with ACE, followed by −7.420 kcal.mol^−1^ for the interaction of VAY peptide with HLA class I histocompatibility antigen A-3 (HLA-A). Overall, APL, IIW, and PVI peptides showed the best cumulative binding energy for the targets, while ACE and inhibitor of apoptosis protein 3 (XIAP) have the best cumulative peptides binding energy. Some of the interactions between the peptides and the molecular targets are shown in Figure 6 and Figure 7.

## 3. Discussion

ALS is a fatal and currently incurable neurodegenerative disease that features the progressive loss of motor neurons in the brainstem, spinal cord, and cerebral cortex [29]. Several ALS-associated proteins are directly involved in the protein quality control (PQC) system, while others indirectly affect the PQC system as a result of mutations [30]. Problems in the PQC forming oligomers have been found to be central in the development of ALS, type II diabetes, Alzheimer’s disease, prion disease, and Parkinson’s disease [31]. Comparison of the published and predicted gene network analyses in this study indicates similarities of YY1 and EGR1 transcription factors, and MAPK14, CDK1, DNAPK, and AKT1 kinases. This work showed that core genes essential for ALS are ATG16L2, SCFD1, VAC15, VEGFA, KEAP1, KIF5A, FIG4, TUBA4A, SIGMAR1, SETX, ANXA11, HNRNPL, NEK1, C9orf72, VCP, RPSA, ATP5B, and SOD1. Other genes including CHRNA3, DAO, GLT8D1, SARM1, ARID1B, G2E3, TMEM175, USP35, PTPRN2, SLC9A8, and MAPKAPK3 seem to be peripheral and less relevant in ALS. It has been reported that some ALS genes are involved in proteostasis (including COPS7A, USP35, TMEM175, and G2E3), mitochondrial metabolism (including ATP5D, ATP5H, and BCS1L) or gene expression and RNA metabolism (including ATXN3, ARID1B, TAF10, and PTBP2), and that decreased expression of NUP50 (a constrained gene encoding a nuclear pore basket protein) was connected with ALS in a transcriptome-wide association study [32]. A recent study of the existing ALS-causing genes has highlighted the role of structural variants in ALS pathogenesis and shown that genomic structural variants in valosin-containing protein (VCP), C9orf72, and Erb-B4 receptor tyrosine kinase 4 (ERBB4) genes impacted the risk of developing ALS, its progression and survival, the site of onset as well as the age of incidence [33].

A study has reported that serotonin receptors help in the positive regulation of TOR signaling, positive regulation of ERK1 and ERK2 cascade, activation of phospholipase C activity, regulation of dopamine secretion, cellular calcium ion homeostasis, and some other biological processes [6]. The role of axonal transport involves the continual supply of cargoes, such as proteins, lipids, RNAs, and organelles required from soma to terminals, and maintains proper neuronal homeostasis through clearance or salvaging of misfolded proteins [34]. Overactivation of mitogen-activated protein kinase (MAPK)/p38 in a mouse model of ALS caused phosphorylation of kinesin-1 and inhibited kinesin-1 translocation along axonal microtubules, leading to transport flaws [35]. A previous study revealed that inhibition of MAPK/p38 could rescue retrograde axonal transport shortcomings [36], whereas inhibition of DNA-dependent protein kinase (DNAPK) biological activities by pharmaceuticals, could restore the axonal transport defects through decrease in the accumulation of phosphorylated FUS in the cytoplasm after DNA impairment resulted from treatment with calicheamicin γ1 [37]. Additionally, mutant SOD1 could cause impairment of the axonal transport possibly through blockage of dynein/dynactin function and might be linked to defective autophagic flux in the motor neurons of ALS [38,39].

In ALS, there is the possibility of several genes fusing with more than one partner [40]. Gene fusion is a rearrangement process that occurs in the chromosome in which two genes (independent) merge to form a new gene (hybrid). Gene fusion usually involves deletion, insertion, translocation, inversion, or read-through transcription of adjacent genes [40]. Recently, several fusion genes have been identified from RNA-Seq data of ALS patients, including TVP23C–CDRT4, AC090517.5–ZNF280D, MAILR–ATP6V1C1 and C8orf44–SGK3 by neighbor fusion as well as YAF2–RYBP by inter-chromosomal fusion [41]. Based on the results of this study, the fusion of DCTN1-KIF5A requires further investigation. Mutations in DCTN1, KIF5A, and ALS2 genes have been implicated as a susceptibility factor for ALS [34,42,43]. An in vitro study has found that oligomerization occurs mostly by KIF5A rather than KIF5B and KIF5C [44]. It has been suggested that some unknown proteins could bind to the abnormal C-terminus of KIF5A (∆exon27) and trigger hyperactivation and aggregation, which changes cellular metabolisms and causes neuronal death [45,46].

Tripeptides are small molecules that have high gastrointestinal absorption, cross the cell membrane, and effect necessary metabolic changes. A previous study has computationally identified CGH peptide, targeted the aggregated mutant SOD1 protein, with higher binding affinity and inhibitory activity [47]. The three enzymes hydrolyzed the selected protein/peptides in this study at F, K, H, L, R, Y, M, N, and W amino acid residues. This makes hydrolysate peptides APVSIPQ and PVI relevant at the C-terminal. However, their presence at the N-terminal will lead to fused peptides.

Peptides AGL and SGGVVK are fragments obtained from calcitonin gene-related peptide 1 (CGRP1, UniProt ID: P06881, seq. 83–119), which contains 128 amino acids and functions as an inducer of vasodilation in many biological vessels such as cerebral, coronary, and systemic vasculature, and its relative abundance in the CNS could defined its role as a neurotransmitter or neuromodulator. Peptide AIF and VAY are fragments obtained from γ-aminobutyric acid receptor-associated protein-like 2 (GABARAPL2; UniProt ID: P60520), which consists of 117 amino acids and functions as ubiquitin-like modifier in autophagy and intra-Golgi traffic, and in mitophagy. GABARAPL2 controls mitochondrial quality and quantity by eradicating the mitochondria to a basal level to achieve cellular energy requirements and prevents excessive ROS production.

The peptide AVK is a fragment obtained from the vasoactive intestinal peptide (VIP) (UniProt ID: P01282, seq.125–152). VIP consists of 170 amino acid residues and functions as a vasodilator, lowers arterial blood pressure, increases glycogenolysis, stimulates myocardial contractility, and relaxes the smooth muscle of the stomach, trachea, and gall bladder. A study has shown that AVK and other peptides could inhibit the interaction between the γ-adaptin subunit of the AP-1 adaptor complex and the kinesin KIF13A, impacting melanogenesis [48].

Peptide APL is obtained from Spadin and has been reported to be one of the peptides derived from the hydrolysis of the skin of Atlantic salmon (*Salmo salar*) and Atlantic herring (*Clupea harengus* L.), with functional roles in the cardiovascular system [49,50]. Spadin is a partial propeptide (Ala12–Arg28) derived from a 44-residue N-terminal propeptide (Gln1-Arg44, propeptide), which is cleaved from sortilin by furin [51]. Sortilin and TREK-1 genes have been implicated in depression due to their high expression in the cerebral structures, and a study has shown that TREK-1 activity was efficiently inhibited by spadin in CA3 hippocampal neurons in brain slices, cultured hippocampal pyramidal neurons, and COS-7 cells [52]. A subchronic treatment of the adult mouse hippocampus with spadin showed that it activated neurogenesis and transcription factor cAMP response element-binding protein (CREB), with direct inhibition of TREK-1, which caused increased neuron excitability during a forced swim test (FST) [53]. Furthermore, CREB mediates transcriptional responses to high levels of cAMP due to a chronic administration of antidepressant, thus triggering downstream targets such as the brain-derived growth factor (BDNF) [54,55].

Peptide APVSIPQ is obtained from NAP peptide (seq.: NAPVSIPQ), a peptide that is part of the sequence of activity-dependent neuroprotective protein (ADNP). NAP peptide can cross the blood-brain barrier (BBB), enhance assembly of microtubules, interact with tubulin, and promote neuronal outgrowth. This peptide also stimulates the MAPK/ERK and PI3-K/Akt pathways, regulates polyADP-ribosylation, and produces extended life span in ALS mouse models [56]. Peptide IGF and IIW are obtained from Peptide H3 (seq.: IGFLIIWV), a transepithelial transported intestinal peptide (obtainable from hydrolytic cleavage of hempseed protein with pepsin), they possesse both anti-inflammatory and antioxidant activities in HepG2 cells [57]. Peptide H3 controls the release of anti-inflammatory cytokines (interleukin 10 (IL-10)), proinflammatory cytokines (tumor necrosis factor (TNF), interferon gamma (IFN-γ), and IL-6), and NO through the regulation of the inducible nitric oxide synthase (iNOS) and NF-κB pathways, exerting an effective anti-inflammatory property [57,58]. IIW peptide has been reported to be one of the products of hydrolytic cleavage of endothelins by neutral endopeptidase and thermolysin [59]. In this present study, the results show that IIW peptide could modulate human endothelin receptor ET-A/ET-B, which is present in the central and peripheral nervous system [60]. Endothelin-1 is richly expressed by reactive astrocytes in the spinal cord of the SOD1^G93A^ mouse model of ALS and in sALS patients, and it has a toxic effect on cultured motor neurons via mechanisms mediated by reactive astrocytes, nitric oxide, and the PI3K/Akt pathway [61,62].

Peptide PVI is obtained from TNKPVI, as a product of in silico simulated digestion of potato patatin by pepsin and trypsin, which greatly lowered the quantity of pro-inflammatory IL-6, IL-8, and TNF in lipopolysaccharide-activated human monocytic (THP-1) cells [63]. Peptide SDPF is obtained from Phoenixin (PNX-14) (seq. 51–64). PNX exists in two forms, 14- and 20-amino acid residues amidated peptides, highly conserved across species, and bind to G protein-coupled receptor (Gpr173). PNX-14 is present in the brain, spinocerebellar tract, spinal trigeminal tract, and cells amid crypts of the small and large intestines, and it is highly co-expressed together with neuropeptide nesfatin-1, which is involved in food intake, glucose homeostasis, and energy expenditure [64,65,66].

Eleven peptides in this study showed at least a 40% probability of targeting particular proteins. Two peptides, IGF and SGGVVK, had lower values and, thus, were considered to be less relevant as potential bioactive agents. Our previous study [20] showed that peptides LPF, AIVF, and PIL could target E3 ubiquitin-protein ligase XIAP (UniProt ID: P98170), peptide GGL could target cyclooxygenase-2 (UniProt ID: P35354), peptides PGF, LPF, AIVF, and QIGLF could target ACE, while peptides PIL, QIGL, and AVL could target DPP4.

Amongst the peptides predicted in this study, the results indicated that AGL has the highest probability of targeting COX-2. Inhibition of COX-2 is beneficial to ALS patients because it limits glutamate release and excitotoxicity. COX-2 is present in spinal neurons and astrocytes and catalyzes reactions that produce prostaglandins, which are inflammatory mediators, specifically, prostaglandin E2 that stimulates glutamate release from astrocytes; its inhibition could significantly reduce astrogliosis and microglial activation [67,68]. Studies have shown that mRNA levels of COX-2 and amount of IL-1β are especially increased in the spinal cord of SOD-1 transgenic mice displaying ALS-type disease [69,70,71,72]. COX-2 inhibition has proven to be effective in limiting neuronal damage in ischemic rodent models, as well as neuroprotective in organotypic spinal cord cultures [67,73,74]. In addition, it is evident that inflammatory mechanism of COX-2 is facilitated by CD40 to promote loss of motor neuron [75].

Molecular hypoventilation could cause failure of respiration in ALS patient, leading to enhanced CO_2_ and reduced O_2_ levels, with the possible occurrence of acidosis-induced death [6,76]. A previous study of TDP-43^A315T^ ALS model and patients, has demonstrated that exposure to O_3_ significantly altered the expression profile of protein kinase B (Akt), signal transducer and activator transcription 3 (STAT3) phosphorylation, and hypothalamic neuropeptides, which led to a significant rise in metabolism and genes involve in thermogenesis [18]. Ozone could activate NF-κB and phosphatases [77], and ozone-driven cellular oxidation has been linked to fatty acid oxidation, acting as a signal traducer for Nrf2/Keap1/ARE system activation with crosstalk of Nrf2/NF-kB and AMPK/FOXO/mTOR/Sir1 pathway, which shifted cytokine activation from pro-inflammatory to anti-inflammatory and immunoregulatory effects on intestinal microbiota and regulatory T lymphocytes [78]. Activation of astrocytes and microglia cause high expression of IL-1β, COX-2, and TNF-α at the onset and during the progression of ALS in SOD1^G93A^ mice, and that treatment with non-steroidal anti-inflammatory drugs (NSAIDs), rofecoxib or celecoxib, markedly inhibited the expression of COX-2 [68,79,80].

In this study, the results indicated that APL, AVK, IIW, and PVI peptides have a high probability of targeting ACE, which is a dipeptidyl peptidase transmembrane enzyme. The ACE inhibitors (ACEIs) have protective effect on ALS, and a dose-dependent inverse association was established between the risk for developing ALS and the use of ACEIs [81]. Previous studies have reported an ACE inhibitory effect of AVL, LVE, LEE, and LVL peptides [82,83,84]. ACEIs are primary agents for the management of hypertension, but a genetic study using Mendelian randomization methods reported that ALS did not cause changes in blood pressure, and that a protective factor for ALS is associated with increased diastolic blood pressure, while an independent risk factor for ALS is associated with increased systolic blood pressure [85]. Moreover, the therapeutic potential of temocapril has been reported in MND such as ALS and neuropathy, through its neurotrophic activity via increase in choline acetyltransferase (ChAT) activity and neurite outgrowth [86].

The results also showed that APL and PVI peptides have a high probability of targeting DPP4. Increases in glucose metabolism is possible in ALS by a strategy that increases pyruvate availability and glucose-6-phosphate hydrogenase activity, with decreased consumption of alternative tricarboxylic acid cycle substrates in the cortex and increase in the level of ribose 5-phosphate and glucose-6-phosphate in the spinal cord of SOD1^G93A^ ALS mice [87]. The incretin hormones glucagon-like peptide-1 (GLP-1) and glucose-dependent insulinotropic polypeptide (GIP) are rapidly metabolized by DPP4 [88]. In mice, oral administration of diapin (Gly-Gly-Leu) peptide was shown to possess a blood-glucose lowering-effect by stimulating GLP-1 secretion from L-cells [89]. DPP4 inhibitors like APL and PVI are potential antidiabetic peptides for lowering glycemic indices, lowering the amount of glucagon, and maintaining insulin levels in type 2 diabetes mellitus [90], and their activities are indirectly facilitated by the continuous presence of GLP-1 incretin [91]. For example, neuroprotective effects by linagliptin involve DPP4 inhibition, which has been linked to peripheral functions in the nervous system due to its inability to permeate the BBB [92,93].

AIF, APL, AVY, and PVI peptides showed high probability of promoting the expression of X-linked inhibitor of apoptosis protein (XIAP). XIAP is the most effective of the IAPs, consisting of two conserved motifs, the zinc finger-like RING finger and baculovirus inhibitory repeats (BIR), and its cleavage produces fragmented peptides that could modulate caspases [94,95]. XIAP has shown to possess neuroprotective effect in ALS through its involvement in the inhibition of caspases to prolong survival and stabilization of the altered calpastatin/calpain system in the ALS mice [96]. The mRNA and protein levels of XIAP were significantly declined in the spinal cord of symptomatic SOD1^G93A^ transgenic mice, and this serves as a potential therapeutic target for fALS [97,98]. Moreover, XIAP enhances the ubiquitination and degradation of COMMD1, a protein that promotes the efflux of copper from the cell. Thus, XIAP can regulate the export of copper from the cell and potentially represents an extra intracellular sensor for copper levels; copper bound XIAP does not prevent caspases activity, and cells that express this form of the protein exhibit increased rates of cell death in response to apoptotic stimuli [99].

However, a study has reported that tissues and cells derived from XIAP-deficient mice contain reduced levels of copper, while inhibition of MURR1 caused increased intracellular copper in cultured cells [100]. A study has identified the copper chaperone for superoxide dismutase (CCS) as a moderator of copper delivery to XIAP in cells [101]. Furthermore, the level of neuronal apoptosis inhibitory protein (NAIP) was lower in ALS and could be upregulated by bromocriptine mesylate (BRC) and WN1316 drug candidates [102,103,104]. Thus, concerted simultaneous inhibition of XIAP-CCS complex formation, and activation of MURR1 in ALS patients by the peptides is a promising effective therapeutic strategy that need urgent research attention.

Permeability glycoprotein (P-gp; ABCB1; MDR1) belongs to the ATP-binding cassette (ABC) superfamily, and it is an active efflux transporter of an extensive range of xenobiotics and endogenous compounds across the cell membrane which requires expenditure of energy in terms of ATP, and it is localized in the gastrointestinal tract, BBB, kidneys, liver, and placenta of humans [105,106]. P-gp efflux and cytochrome P450s (CYPs) activities can greatly impact drug pharmacokinetics by clinically affecting the drug effectiveness, due to the fact that there are more than 55 CYP homologues in humans of which 90% of therapeutic ingredients are metabolized by CYP1A2, CYP2E1, CYP2C19, CYP2C9, CYP2D6, CYP3A5, and/or CYP3A4, [106]. In the ALS *SOD1*^G93A^ mouse model, damages in the BBB and micro-vessels of post-mortem spinal cord and brain tissues have been reported [107]. ALS, epilepsy, and stroke are typical neurologic diseases associated with an overexpression of P-gp in the BBB endothelial cells, this prevents penetration of drugs through the tissues present in the CNS and is affected by the downregulation in major tight junction proteins [108]. A study has shown that upregulation of P-gp probably reduced the loss of P-gp substrates across a dysfunctional BBB in an ALS model [109]. Furthermore, the allergenicity prediction results excluded peptides AVY and SDPF as they are probable allergens, whereas AGL, APL, AVK, IIW, PVI, and AIF peptides were forecasted to be non-allergenic negative modulators of P-gp, making them strong candidates for further investigation.

Molecular docking predicts ligand binding sites (active and allosteric/regulatory sites) on biological macromolecules (proteins, RNA, or DNA) surfaces and predictively calculates the binding affinities of their interaction [110]. The binding energy score of ≤−5.00 kcal.mol^−1^ indicates good affinity between the target protein and the ligand [111]. The interaction of VAY peptide with OPRM1 shows weak interaction as it is >−5.00 kcal.mol^−1^; however, AGL, AIF, APL, AVK, IIW, and PVI are the most promising peptides as their binding energy scores are ≤−5.00 kcal.mol^−1^. A previous study on the molecular docking of mutant SOD1 with tripeptide CGH reported an overall binding affinity of −5.35 kcal.mol^−1^ [47]. Additionally, peptides PGF, AVL, and AIVF could bind to COX-2 and XIAP with binding affinities between −6.50 and −8.30 kcal.mol^−1^ [20], which support their potential use as modulators of reactions involved in ALS pathology.

## 4. Materials and Methods

### 4.1. Preparation of ALS-Associated Genes

The genes implicated in ALS in humans and animal models were adapted from the literature without duplication, yielding a total of 76 genes. Forty eight genes (*ATXN2, ANXA11, ALS2, ATXN3, ANG, C9orf72, CHMP2B, CHRNA3, CHCHD10, DCTN1, DNAJC7, DAOEWSR1, ERBB4, FUS, FIG4, GLT8D1, GLE1, hnRNPA2B1, hnRNPA1, KIF5A, MATR3, NEK1, NIPA1, NEFH, OPTN, PON1, PON2, PON3, PFN1, PRPH, SOD1, SETX, SQSTM1, SARM1, SPG11, SIGMAR1, TARDBP, TBK1, TUBA4A, TIA1, TAF15, UNC13A, UBQLN2, VCP, VAPB, VEGF,* and *WDR7*) were collected from Smukowski et al. [8], 11 genes (*ARID1B, ATP5H, ATP5D, BCS1L, COPS7A, G2E3, NUP50, PTBP2, TAF10, TMEM175,* and *USP35*) from Megat et al. [32], 11 genes (*CFAP410*, *COG3*, *ERGIC1*, *GPX3, TNIP1*, *HLA, MOBP, RPSA*, *PTPRN2*, *SLC9A8,* and *SPATA2*) from van Rheenen et al. [112], and 6 genes (*ACSL5*, *ATG16L2*, *MAPKAPK3*, *MAP1LC3A*, *SCFD12*, and *PLXNB*) from Saez-Atienzar et al. [1].

### 4.2. Target Genes Network Analysis

All 76 ALS-associated published genes were used for network analysis to determine the enrichment of transcription factors and kinases as well as protein-protein interaction. This was carried out on the eXpression2Kinases (X2K) webserver, available at https://maayanlab.cloud/X2K/ [113] (accessed on 23 September 2022), and human (*Homo sapiens*) was designated as the target organism.

### 4.3. Protein-Protein Interaction

The protein-protein interaction (PPI) profile of the 76 ALS-associated genes were obtained from the STRING webserver (https://string-db.org/, [114]) (accessed on 23 September 2022), revealing the gene network and gene fusions.

### 4.4. Protein Hydrolysis and Bioactivity Assessment

Several peptides that have been reported with suitable biological activity such as antioxidant, anti-neurodegeneration, antidiabetic, and anti-inflammatory in ALS studies were selected for this analyses, these include: P5-Best [115]; peptide H3 [58]; NAP, ADNF-9, and humanin [116]; soy-deprestatin [117]; spadin [52,53]; pro-neuropeptide Y (PNY) (seq.30–64), calcitonin gene-related peptide 1 (CGP)(seq.83–119), substance P (SP) (seq.1–11), vasoactive intestinal peptide (VIP) (seq.125–152), proenkephalin-A (PEA) (seq.209–237), α-melanocyte-stimulating hormone (α-MSH) (seq.1–13), and phoenixin (PNX-14) all from Wei et al. [66]; and LAVYPWT (as standard neuropeptides obtained from BIOPEP-UMW) (supplementary Table S1) were obtained from various online databases and literature. The sequences were subjected to simulate enzymatic hydrolysis on the BIOPEP-UWM webserver using the combination of digestive enzymes chymotrypsin (EC 3.4.21.1), pepsin, pH 1.3 (EC 3.4.23.1), and trypsin (EC 3.4.21.4) [118]. The hydrolysate peptide sequences were converted to simplified molecular input line entry specification (SMILES) format on the BIOPEP-UWM webserver prior to further analysis, as previously reported [20].

### 4.5. Target Prediction and Reconstruction of Gene Network

The sequence of hydrolysate peptides obtained from the in silico enzymatic hydrolysis (supplementary Table S1) were used for the target prediction analysis on the SwissTargetPrediction (http://www.swisstargetprediction.ch, accessed on 25 September 2022), human (*Homo sapiens*) was designated as the target organism [119], and a threshold of 20% probability was used as the selection criteria. The predicted gene IDs were then used for another network analyses to determine the enrichment of transcription factors and kinases as well as protein-protein interactions. This was carried out on the eXpression2Kinases (X2K) webserver, available at https://maayanlab.cloud/X2K/ [113] (accessed on 25 September 2022), and human (*Homo sapiens*) was designated as the target organism.

### 4.6. In Silico ADME and Allergenicity Prediction

The in silico ADME (absorption, distribution, metabolism, and excretion) screening for the functional hydrolysate peptides was done on the SwissADME server (www.swissadme.ch, accessed on 25 September 2022) [120], which was performed at the default parameters using the SMILES format. The estimation of allergenicity was carried out on the AllerTOP v.2.0 webserver; http://www.ddg-pharmfac.net/AllerTOP, accessed on 25 September 2022) [121].

### 4.7. Molecular Docking Studies

The criteria used for the molecular docking studies are peptides with at most four amino acid residues [110,122,123], are non-allergenic, and have a minimum of 40% probability of target on at least one molecular target protein. Based on the predicted molecular targets obtained in the previous step, peptide-protein docking was carried out on the HPEPDOCK webserver (https://huanglab.phys.hust.edu.cn/hpepdock, accessed on 30 September 2022) [124], at default the setting, as previously reported [20]. In addition, peptide-protein molecular docking was carried out using AutoDock Vina v1.2.3 [125,126], as previously reported [6]. Prior to docking, the peptides’ (ligands’) target proteins were formatted to pdbqt by using the AutoDock Tools (ADT) v1.5.6 [127]. Afterward, binding affinity was obtained and close interactions of binding of the ligands with the target were visualized on the PoseView webserver, available at https://proteins.plus/ [128] (accessed on 3 October 2022).

## 5. Conclusions

This study computationally identified unique sets of peptides from in silico enzyme hydrolysis of proteins that could serve as therapeutic agents against ALS based on strong evidence that supports targeting multi-metabolic components in disease pathogenesis. A typical novel unfused peptide that could be designed is AGLAIFAPLAVKIIWVAYPVI (21 sequence length), as this novel peptide could be cleaved by the gut hydrolytic enzymes into the desired fragmented active peptides. Overall, results from pharmacokinetics, molecular target predictions, and binding affinity indicated that the tripeptides AGL, APL, AVK, IIW, PVI, and VAY are the most promising candidates for further study. These peptides could synergistically serve as potential effective anti-ALS agents in combination with some supplements such as D-3β-hydroxybutyrate and trehalose. The future work would validate the therapeutic properties of these hydrolysate peptides by in vitro and in vivo approaches using cell-based and animal models of ALS disease.

## Figures and Tables

**Table 1:** Molecular targets of hydrolysate peptides predicted from SwissTargetPrediction.

	Selected Active Hydrolysate Peptide	% Probability of Predicted Targets																						
SN		A	B	C	D	E	F	G	H	I	J	K	L	M	N	O	P	Q	R	S	T	U	V	W
1	AGL	65	20	20	20	20																		
2	AIF	25	25	20			55	35	25	20	20													
3	APL		80	20	75	25	50					20												
4	APVSIPQ			35			30						20											
5	AVK		45	25	20	20								20	20	20								
6	AVY		35			20	40	50	50		30						25							
7	IGF	30	20				30																	
8	IIW		45					40	25									50	30	20				
9	PVI	20	50	20	80		80																	
10	SDPF			20									20								45	30	25	25
11	SGGVVK			20		20									45	20								
12	VAY	20		40		20	35	45	45	20	30						25							

Note: Target proteins name (Gene ID at www.genecards.org (accessed on 25 September 2022), UniProt ID at www.uniprot.org (accessed on 25 September 2022)). **A:** cyclooxygenase-2 (PTGS2, P35354). **B:** angiotensin I-converting enzyme (ACE, P12821). **C:** HLA class I histocompatibility antigen A-3 (HLA-A, P04439). **D:** dipeptidyl peptidase IV (DPP4, P27487). **E:** Calpain 1 (CAPN1, P07384). **F:** inhibitor of apoptosis protein 3 (XIAP, P98170). **G:** mu opioid receptor (OPRM1, P35372). **H:** delta opioid receptor (OPRD1, P41143). **I:** Neprilysin (MME, P08473). **J:** kappa opioid receptor (OPRK1, P41145). **K:** leucine aminopeptidase (LAP3, P28838). **L:** disks large homolog 4 (DLG4, P78352). **M:** cathepsin (B and K) (CTSB, P07858). **N:** lipoxin A4 receptor (by homology) (FPR2, P25090). **O:** NAD-dependent deacetylase sirtuin 1 (SIRT1/SIRT2, Q96EB6/Q8IXJ6). **P:** tyrosyl-tRNA synthetase (YARS, P54577). **Q:** endothelin receptor ET-A/ET-B (EDNRA/EDNRB, P25101/P24530). **R**: neurokinin 1 receptor (TACR1, P25103). **S:** cholecystokinin B receptor (CCKBR, P32239). **T:** HMG-CoA reductase (HMGCR, P04035). **U:** calcitonin gene-related peptide type 1 receptor (CALCRL, Q16602). **V:** beta-secretase 1 (BACE1, P56817). **W**: ribonucleoside-diphosphate reductase M1 chain (RRM1, P23921).

**Table 2:** Predicted pharmacokinetic properties of the hydrolysate peptides.

SN	Hydrolysate Peptides (Ligands)	Predicted ADME Parameter													
		MW	MR	TPSA (Å^2^)	Log P	ESOL Log S	ESOL Class	GIA	BBB	P-gp	CYPs Inhibitor	Log Kp (cm/s)	BS	SA	Allergenicity
1	AGL	259.3	65.47	121.52	−0.76	0.99	Highly soluble	High	No	No	None	−9.95	0.55	2.69	Non-allergen
2	AIF	349.42	94.77	121.52	0.56	−0.4	Very soluble	High	No	No	None	−9.62	0.55	3.49	Non-allergen
3	APL	299.37	81.79	112.73	−0.37	0.65	Highly soluble	High	No	Yes	None	−10.17	0.55	3.21	Non-allergen
4	APVSIPQ	710.82	186.29	283.66	−1.48	−0.24	Very soluble	Low	No	No	None	−13.36	0.17	6.34	Non-allergen
5	AVK	316.4	82.6	147.54	−0.83	1.74	Highly soluble	Low	No	No	None	−11.33	0.55	3.41	Non-allergen
6	AVY	351.4	91.98	141.75	−0.18	0.33	Highly soluble	Low	No	No	None	−10.55	0.55	3.27	Allergen
7	IGF	335.4	89.96	121.52	0.51	−0.41	Very soluble	High	No	No	None	−9.43	0.55	3.19	Non-allergen
8	IIW	430.54	121.05	137.31	1.82	−1.98	Very soluble	High	No	Yes	CYP3A4	−8.8	0.55	4.32	Non-allergen
9	PVI	327.42	91.4	107.53	0.35	−0.04	Very soluble	High	No	Yes	None	−9.61	0.55	3.58	Non-allergen
10	SDPF	464.47	117.02	199.36	−1.72	1.4	Highly soluble	Low	No	No	None	−12.89	0.11	4.14	Allergen
11	SGGVVK	545.63	136.03	255.07	−1.88	1.09	Highly soluble	Low	No	Yes	None	−12.77	0.17	4.89	Non-allergen
12	VAY	351.4	91.98	141.75	−0.1	0.21	Highly soluble	Low	No	No	None	−10.41	0.55	3.27	Allergen

SN: Serial number. Legend: Physicochemical properties: molecular weight (MW), molar refractivity (MR), total polar surface area (TPSA). Lipophilicity: log P (consensus). Water solubility: ESOL log S, ESOL class. Pharmacokinetics: gastrointestinal absorption (GIA), blood-brain barrier (BBB), P-glycoprotein (P-gp) substrate, inhibition of cytochrome P450 (CYPs) type CYP1A2, CYP2C19, CYP2C9, CYP2D6, and CYP3A4, skin permeation (Log Kp). Drug-likeness: bioavailability score (BS). Medicinal chemistry: synthetic accessibility (SA).

**Table 3:** Docking binding affinity score of the interaction between ligands from HPEPDOCK Server and AutoDock Vina.

**Selected Active Hydrolysate Peptides**	**HPEPDOCK Docking Score**							
	**A**	**B**	**C**	**D**	**F**	**G**	**H**	**Q**
AGL	−91.468							
AIF					−116.310			
APL		−100.047		−112.268	−91.378			
AVK		−99.983						
IIW		−172.819				−188.427		−147.996
PVI		−128.131		−118.817	−119.050			
VAY			−137.310			−144.350	−114.143	
**Selected Active Hydrolysate Peptides**	**AutoDock Vina Binding Affinity (kcal.mol^−1^)**							
	**A**	**B**	**C**	**D**	**F**	**G**	**H**	**Q**
AGL	−5.462							
AIF					−6.723			
APL		−6.909		−5.380	−5.734			
AVK		−5.679						
IIW		−7.912				−6.660		−6.897
PVI		−5.083		−5.832	−5.194			
VAY			−7.420			−4.452	−5.988	

Target proteins name (Gene ID at www.genecards.org (accessed on 25 September 2022), UniProt ID at www.uniprot.org (accessed on 25 September 2022)). **A:** cyclooxygenase-2 (PTGS2, P35354). **B:** angiotensin I-converting enzyme (ACE, P12821). **C:** HLA class I histocompatibility antigen A-3 (HLA-A, P04439). **D:** dipeptidyl peptidase IV (DPP4, P27487). **F:** inhibitor of apoptosis protein 3 (XIAP, P98170). **G:** mu opioid receptor (OPRM1, P35372). **H:** delta opioid receptor (OPRD1, P41143). **Q:** endothelin receptor ET-A (EDNRA, P25101).

## Data Availability

The data from the current study are available from the corresponding author and first author of the article upon reasonable request.

## Supplementary Materials

The following supporting information can be downloaded at: https://www.mdpi.com/article/10.3390/ijms24065828/s1.
