# Supplementary material for: In Silico Exploration of Metabolically Active Peptides as Potential Therapeutic Agents against Amyotrophic Lateral Sclerosis
Source: Int J Mol Sci. 2023 Mar 18;24(6):5828. doi: 10.3390/ijms24065828 (PMC10058213; doi:10.3390/ijms24065828)
Supplement: Supplementary file 1 [file ijms-24-05828-s001.zip › ijms-2223476-supplementary.pdf]

Supplementary Table 1: The insilico hydrolysis of the investigated peptides and proteins by chymotrypsin, trypsin and pepsin simultaneous cleavage.

**PEPTIDE NAME**	**SEQUENCE and hydrolysate from chymotrypsin, trypsin and pepsin simultaneous treatment by insilico**	**Selected hydrolysate***	**SMILES**
P5-Best	YLPKHSDRD<br><br>Y - L - PK - H - SDR - D	SDR	N[C@@]([H])(CO)C(=O)N[C@@]([H])(CC(=O)O)C(=O)N[C@@]([H])(CCCNC(=N)N)C(=O)O
Peptide H3	IGFLIIWV<br><br>IGF - L - IIW - V	**IGF**	N[C@@]([H])([C@]([H])(CC)C)C(=O)NCC(=O)N[C@@H](CC1=CC=C(C=C1))C(=O)O
		**IIW**	N[C@@]([H])([C@]([H])(CC)C)C(=O)N[C@@]([H])([C@]([H])(CC)C)C(=O)N[C@@H](CC1=C[NH]C2=CC=CC=C12)C(=O)O
NAP	NAPVSIPQ<br><br>N - APVSIPQ	APVSIPQ	N[C@@]([H])(C)C(=O)N1[C@@]([H])(CCC1)C(=O)N[C@@]([H])(C(C)C)C(=O)N[C@@]([H])(CO)C(=O)N[C@@]([H])([C@]([H])(CC)C)C(=O)N1[C@@]([H])(CCC1)C(=O)N[C@@]([H])(CCC(=O)N)C(=O)O
ADNF-9	SALLRSIPA<br><br>SAL - L - R - SIPA	SAL	N[C@@]([H])(CO)C(=O)N[C@@]([H])(C)C(=O)N[C@@]([H])(CC(C)C)C(=O)O
		SIPA	N[C@@]([H])(CO)C(=O)N[C@@]([H])([C@]([H])(CC)C)C(=O)N1[C@@]([H])(CCC1)C(=O)N[C@@]([H])(C)C(=O)O
Humanin	MAPRGFSCLLLLTSEIDLPVKRRA<br><br>M - APR - GF - SCL - L - L - L - TSEIDL - PVK - R - R - A	APR	N[C@@]([H])(C)C(=O)N1[C@@]([H])(CCC1)C(=O)N[C@@]([H])(CCCNC(=N)N)C(=O)O
		SCL	N[C@@]([H])(CO)C(=O)N[C@@]([H])(CS)C(=O)N[C@@]([H])(CC(C)C)C(=O)O
		TSEIDL	N[C@@]([H])([C@]([H])(O)C)C(=O)N[C@@]([H])(CO)C(=O)N[C@@]([H])(CCC(=O)O)C(=O)N[C@@]([H])([C@]([H])(CC)C)C(=O)N[C@@]([H])(CC(=O)O)C(=O)N[C@@]([H])(CC(C)C)C(=O)O
		PVK	N1[C@@]([H])(CCC1)C(=O)N[C@@]([H])(C(C)C)C(=O)N[C@@]([H])(CCCCN)C(=O)O
Spadin	YAPLPRWSGPIGVSWGLR<br><br>Y - APL - PR - W - SGPIGVSW - GL – R	**APL**	N[C@@]([H])(C)C(=O)N1[C@@]([H])(CCC1)C(=O)N[C@@]([H])(CC(C)C)C(=O)O
		SGPIGVSW	N[C@@]([H])(CO)C(=O)NCC(=O)N1[C@@]([H])(CCC1)C(=O)N[C@@]([H])([C@]([H])(CC)C)C(=O)NCC(=O)N[C@@]([H])(C(C)C)C(=O)N[C@@]([H])(CO)C(=O)N[C@@H](CC1=C[NH]C2=CC=CC=C12)C(=O)O
soy-deprestatin	LSSTQAQQSY<br><br>L – SSTQAQQSY	SSTQAQQSY	N[C@@]([H])(CO)C(=O)N[C@@]([H])(CO)C(=O)N[C@@]([H])([C@]([H])(O)C)C(=O)N[C@@]([H])(CCC(=O)N)C(=O)N[C@@]([H])(C)C(=O)N[C@@]([H])(CCC(=O)N)C(=O)N[C@@]([H])(CCC(=O)N)C(=O)N[C@@]([H])(CO)C(=O)N[C@@H](CC1=CC=C(C=C1)O)C(=O)O

Pro-neuropeptide Y (PNY) (seq.30–64),	PSKPDNPGEDAPAEDMARYYSALRHYINLITRQRY<br>PSK - PDN - PGEDAPAEDM - AR - Y - Y - SAL - R - H - Y - IN - L - ITR - QR - Y	PSK	N1[C@@]([H])(CCC1)C(=O)N[C@@]([H])(CO)C(=O)N[C@@]([H])(CCCCN)C(=O)O
		PDN	N1[C@@]([H])(CCC1)C(=O)N[C@@]([H])(CC(=O)O)C(=O)N[C@@]([H])(CC(=O)N)C(=O)O
		PGEDAPAEDM	N1[C@@]([H])(CCC1)C(=O)NCC(=O)N[C@@]([H])(CCC(=O)O)C(=O)N[C@@]([H])(CC(=O)O)C(=O)N[C@@]([H])(C)C(=O)N1[C@@]([H])(CCC1)C(=O)N[C@@]([H])(C)C(=O)N[C@@]([H])(CCC(=O)O)C(=O)N[C@@]([H])(CC(=O)O)C(=O)N[C@@]([H])(CCSC)C(=O)O
		SAL	N[C@@]([H])(CO)C(=O)N[C@@]([H])(C)C(=O)N[C@@]([H])(CC(C)C)C(=O)O
		ITR	N[C@@]([H])([C@]([H])(CC)C)C(=O)N[C@@]([H])([C@]([H])(O)C)C(=O)N[C@@]([H])(CCCNC(=N)N)C(=O)O
Calcitonin gene-related peptide 1 (CGP)(seq.83–119),	CDTATCVTHRLAGLLSRSGGVVKNNFVPTNVGSKAF<br>CDTATCVTH - R - L - AGL - L - SR - SGGVVK - N - N - F - VPTN - VGSK - AF	CDTATCVTH	N[C@@]([H])(CS)C(=O)N[C@@]([H])(CC(=O)O)C(=O)N[C@@]([H])([C@]([H])(O)C)C(=O)N[C@@]([H])(C)C(=O)N[C@@]([H])([C@]([H])(O)C)C(=O)N[C@@]([H])(CS)C(=O)N[C@@]([H])(C(C)C)C(=O)N[C@@]([H])([C@]([H])(O)C)C(=O)N[C@@H](CC1=CN=C[NH]1)C(=O)O
		**AGL**	N[C@@]([H])(C)C(=O)NCC(=O)N[C@@]([H])(CC(C)C)C(=O)O
		**SGGVVK**	N[C@@]([H])(CO)C(=O)NCC(=O)NCC(=O)N[C@@]([H])(C(C)C)C(=O)N[C@@]([H])(C(C)C)C(=O)N[C@@]([H])(CCCCN)C(=O)O
		VPTN	N[C@@]([H])(C(C)C)C(=O)N1[C@@]([H])(CCC1)C(=O)N[C@@]([H])([C@]([H])(O)C)C(=O)N[C@@]([H])(CC(=O)N)C(=O)O
		VGSK	N[C@@]([H])(C(C)C)C(=O)NCC(=O)N[C@@]([H])(CO)C(=O)N[C@@]([H])(CCCCN)C(=O)O
Vasoactive intestinal peptide (VIP) (seq.125–152),	HSDAVFTDNYTRLRKQMAVKKYLNSILN<br>H - SDAVF - TDN - Y - TR - L - R - K - QM - AVK - K - Y - L - N - SIL – N	SDAVF	N[C@@]([H])(CO)C(=O)N[C@@]([H])(CC(=O)O)C(=O)N[C@@]([H])(C)C(=O)N[C@@]([H])(C(C)C)C(=O)N[C@@H](CC1=CC=C(C=C1))C(=O)O
		TDN	N[C@@]([H])([C@]([H])(O)C)C(=O)N[C@@]([H])(CC(=O)O)C(=O)N[C@@]([H])(CC(=O)N)C(=O)O
		**AVK**	N[C@@]([H])(C)C(=O)N[C@@]([H])(C(C)C)C(=O)N[C@@]([H])(CCCCN)C(=O)O
		SIL	N[C@@]([H])(CO)C(=O)N[C@@]([H])([C@]([H])(CC)C)C(=O)N[C@@]([H])(CC(C)C)C(=O)O
α-Melanocyte-stimulating hormone (α-MSH) (seq.1–13),	MPRSCCSRSGALL<br>M - PR - SCCSR - SGAL - L	SCCSR	N[C@@]([H])(CO)C(=O)N[C@@]([H])(CS)C(=O)N[C@@]([H])(CS)C(=O)N[C@@]([H])(CO)C(=O)N[C@@]([H])(CCCNC(=N)N)C(=O)O
		SGAL	N[C@@]([H])(CO)C(=O)NCC(=O)N[C@@]([H])(C)C(=O)N[C@@]([H])(CC(C)C)C(=O)O
Proenkephalin-A (PEA) (seq.209–237)	RYGGFMRRVGRPEWWMDYQKRYGGFLKRF<br>R - Y - GGF - M - R - R - VGR - PEW - W - M - DY - QK - R - Y - GGF - L - K - R - F	GGF	NCC(=O)NCC(=O)N[C@@H](CC1=CC=C(C=C1))C(=O)O
		VGR	N[C@@]([H])(C(C)C)C(=O)NCC(=O)N[C@@]([H])(CCCNC(=N)N)C(=O)O
		PEW	N1[C@@]([H])(CCC1)C(=O)N[C@@]([H])(CCC(=O)O)C(=O)N[C@@H](CC1=C[NH]C2=CC=CC=C12)C(=O)O
		GGF	NCC(=O)NCC(=O)N[C@@H](CC1=CC=C(C=C1))C(=O)O

Phoenixin (PNX-14) 51-64	DVQPPGLKVWSDPF<br><br>DVQPPGL - K - VW - SDPF	DVQPPGL	N[C@@]([H])(CC(=O)O)C(=O)N[C@@]([H])(C(C)C)C(=O)N[C@@]([H])(CCC(=O)N)C(=O)N1[C@@]([H])(CCC1)C(=O)N1[C@@]([H])(CCC1)C(=O)NCC(=O)N[C@@]([H])(CC(C)C)C(=O)O
		**SDPF**	N[C@@]([H])(CO)C(=O)N[C@@]([H])(CC(=O)O)C(=O)N1[C@@]([H])(CCC1)C(=O)N[C@@H](CC1=CC=C(C=C1))C(=O)O
potato patatin-derived peptide	TNKPVI<br><br>TN - K - PVI	**PVI**	N1[C@@]([H])(CCC1)C(=O)N[C@@]([H])(C(C)C)C(=O)N[C@@]([H])([C@]([H])(CC)C)C(=O)O
VCP region (797-806)	TEDNDDDLYG<br><br>TEDN - DDDL - Y – G	TEDN	N[C@@]([H])([C@]([H])(O)C)C(=O)N[C@@]([H])(CCC(=O)O)C(=O)N[C@@]([H])(CC(=O)O)C(=O)N[C@@]([H])(CC(=O)N)C(=O)O
		DDDL	N[C@@]([H])(CC(=O)O)C(=O)N[C@@]([H])(CC(=O)O)C(=O)N[C@@]([H])(CC(=O)O)C(=O)N[C@@]([H])(CC(C)C)C(=O)O
VCP region (521-526)	GCGKTL<br><br>GCGK – TL	GCGK	NCC(=O)N[C@@]([H])(CS)C(=O)NCC(=O)N[C@@]([H])(CCCCN)C(=O)O
GABARAPL2 peptide	MKWMFKEDHSLEHRCVESAKIR<br>AKYPDRVPVIVEKVSGSQIVDIDK<br>RKYLVPSDITVAQFMWIIRKRIQL<br>PSEKAIFLFVDKTVPQSSLTMGQL<br>YEKEKDEDGFLYVAYSGENTFGF<br><br>M - K - W - M - F - K - EDH - SL - EH - R - CVESAK - IR - AK - Y - PDR - VPVIVEK - VSGSQIVDIDK - R - K - Y - L - VPSDITVAQF - M - W - IIR - K - R - IQL - PSEK - AIF - L - F - VDK - TVPQSSL - TM - GQL - Y - EK - EK - DEDGF - L - Y - VAY - SGEN - TF – GF	EDH	N[C@@]([H])(CCC(=O)O)C(=O)N[C@@]([H])(CC(=O)O)C(=O)N[C@@H](CC1=CN=C[NH]1)C(=O)O
		CVESAK	N[C@@]([H])(CS)C(=O)N[C@@]([H])(C(C)C)C(=O)N[C@@]([H])(CCC(=O)O)C(=O)N[C@@]([H])(CO)C(=O)N[C@@]([H])(C)C(=O)N[C@@]([H])(CCCCN)C(=O)O
		PDR	N1[C@@]([H])(CCC1)C(=O)N[C@@]([H])(CC(=O)O)C(=O)N[C@@]([H])(CCCNC(=N)N)C(=O)O
		VPVIVEK	N[C@@]([H])(C(C)C)C(=O)N1[C@@]([H])(CCC1)C(=O)N[C@@]([H])(C(C)C)C(=O)N[C@@]([H])([C@]([H])(CC)C)C(=O)N[C@@]([H])(C(C)C)C(=O)N[C@@]([H])(CCC(=O)O)C(=O)N[C@@]([H])(CCCCN)C(=O)O
		VSGSQIVDIDK	N[C@@]([H])(C(C)C)C(=O)N[C@@]([H])(CO)C(=O)NCC(=O)N[C@@]([H])(CO)C(=O)N[C@@]([H])(CCC(=O)N)C(=O)N[C@@]([H])([C@]([H])(CC)C)C(=O)N[C@@]([H])(C(C)C)C(=O)N[C@@]([H])(CC(=O)O)C(=O)N[C@@]([H])([C@]([H])(CC)C)C(=O)N[C@@]([H])(CC(=O)O)C(=O)N[C@@]([H])(CCCCN)C(=O)O
		VPSDITVAQF	N[C@@]([H])(C(C)C)C(=O)N1[C@@]([H])(CCC1)C(=O)N[C@@]([H])(CO)C(=O)N[C@@]([H])(CC(=O)O)C(=O)N[C@@]([H])([C@]([H])(CC)C)C(=O)N[C@@]([H])([C@]([H])(O)C)C(=O)N[C@@]([H])(C(C)C)C(=O)N[C@@]([H])(C)C(=O)N[C@@]([H])(CCC(=O)N)C(=O)N[C@@H](CC1=CC=C(C=C1))C(=O)O
		IIR	N[C@@]([H])([C@]([H])(CC)C)C(=O)N[C@@]([H])([C@]([H])(CC)C)C(=O)N[C@@]([H])(CCCNC(=N)N)C(=O)O
		IQL	N[C@@]([H])([C@]([H])(CC)C)C(=O)N[C@@]([H])(CCC(=O)N)C(=O)N[C@@]([H])(CC(C)C)C(=O)O
		PSEK	N1[C@@]([H])(CCC1)C(=O)N[C@@]([H])(CO)C(=O)N[C@@]([H])(CCC(=O)O)C(=O)N[C@@]([H])(CCCCN)C(=O)O
		**AIF**	N[C@@]([H])(C)C(=O)N[C@@]([H])([C@]([H])(CC)C)C(=O)N[C@@H](CC1=CC=C(C=C1))C(=O)O

		VDK	N[C@@]([H])(C(C)C)C(=O)N[C@@]([H])(CC(=O)O)C(=O)N[C@@]([H])(CCCCN)C(=O)O
		TVPQSSL	N[C@@]([H])([C@]([H])(O)C)C(=O)N[C@@]([H])(C(C)C)C(=O)N1[C@@]([H])(CCC1)C(=O)N[C@@]([H])(CCC(=O)N)C(=O)N[C@@]([H])(CO)C(=O)N[C@@]([H])(CO)C(=O)N[C@@]([H])(CC(C)C)C(=O)O
		GQL	NCC(=O)N[C@@]([H])(CCC(=O)N)C(=O)N[C@@]([H])(CC(C)C)C(=O)O
		DEDGF	N[C@@]([H])(CC(=O)O)C(=O)N[C@@]([H])(CCC(=O)O)C(=O)N[C@@]([H])(CC(=O)O)C(=O)NCC(=O)N[C@@H](CC1=CC=C(C=C1))C(=O)O
		**VAY**	N[C@@]([H])(C(C)C)C(=O)N[C@@]([H])(C)C(=O)N[C@@H](CC1=CC=C(C=C1)O)C(=O)O
		SGEN	N[C@@]([H])(CO)C(=O)NCC(=O)N[C@@]([H])(CCC(=O)O)C(=O)N[C@@]([H])(CC(=O)N)C(=O)O
TUBA4A (142-148)	GGGTGSG	GGGTGSG	NCC(=O)NCC(=O)NCC(=O)N[C@@]([H])([C@]([H])(O)C)C(=O)NCC(=O)N[C@@]([H])(CO)C(=O)NCC(=O)O
BIOPEP-UWM	LAVYPWT<br><br>L - AVY - PW - T	**AVY**	N[C@@]([H])(C)C(=O)N[C@@]([H])(C(C)C)C(=O)N[C@@H](CC1=CC=C(C=C1)O)C(=O)O

*Selected Active peptides are in bold
